# Downstream assessment of chlorinated organic compounds in the bed-sediment of Aiba Stream, Iwo, South-Western, Nigeria

**DOI:** 10.1186/s40064-016-1664-0

**Published:** 2016-01-22

**Authors:** Godwin O. Olutona, Stephen O. Olatunji, Joshua F. Obisanya

**Affiliations:** Department of Chemistry and Industrial Chemistry, Bowen University, P. M. B. 284, Iwo, Nigeria; Department of Chemistry, Cape Peninsula University of Technology, Cape Town, South Africa; Institute for Entrepreneurship and Development Studies, Obafemi Awolowo University, Ile-Ife, Nigeria

**Keywords:** Organochlorine pesticides, Sediment, Stream, Contamination

## Abstract

This study investigated levels and distribution pattern of chlorinated organic compounds (COCs) otherwise known as organochlorine pesticides in sediment samples at downstream of Aiba watercourse in Iwo, South-western Nigeria. Soxhlet extraction method followed by GC–ECD analysis were used to ascertain levels of COCs in the sediment samples collected from four different locations along the stream. Eighteen COCs were detected with trans permethrin and endosulfan sulfate having highest and lowest concentrations of 375.70 ± 689.41 and 0.03 ± 0.05 µg/g, respectively. The varying levels of COCs as obtained in this study were attributed to organochlorine pesticides contamination emanated from different agricultural practices and domestic sewage loads of the study area.

## Background

Chlorinated organic compounds (COCs) otherwise known as organochlorine pesticides (OCPs) is one of the multi-arrays of hydrophobic organic compounds which have been produced in large quantity, and used for the control of weeds, termites, mosquitoes and other insects constituting nuisance in many part of the world for years (Ssebugere et al. 2013, 2014). Pesticides are partitioned between air, water and soil during application or after application. Pesticides may also enter aquatic systems via atmospheric deposition, soil erosion and sewage runoff, industrial and agricultural runoff (Yang et al. 2005). Concern about COCs is as a result of their lipophilic attributes, low susceptibility to biodegradation, chemical stability and persistence, bio-magnification and accumulation tendency in the food chain, long and wide range distribution and atmospheric transport in the environment and their potential negative impact to both aquatic habitat and humans (Basheer et al. 2002).

Water is a major pathway through which pesticides are transported from area of primary application to other compartments within the environment, especially the aquatic system. COCs could spread into aquatic environments through runoffs from non-point soil source (Aly Salem et al. 2013). Gilliom (2007) reported that pesticides; particularly those used for agriculture were often detected, and at highest concentrations in stream traversing agricultural area. Once in water, pesticides are further distributed between biotic and abiotic components, such as plants, fishes, benthic organism and sediments. Sediment is a matrix of materials which is comprised of detritus, inorganic and organic particles and is relatively herterogeneous in terms of physical, chemical and biological characteristics (Hakanson 1992). Zheng et al. (2009) underscored roles of sediment as a secondary source of COCs in the environment due to its substantial retention capacity of the organic pollutants which might be re-emitting under favourable conditions into the ecosystem. Hence, it determines distribution and fate of COCs in aquatic environment. In the same vein, Brasher and Anthony (2000) regarded sediment as sink, removal agent and vehicle for a host of contaminants in marine environs. Consequently, stream’s bed sediment of the study area could be more susceptible to high concentrations of COCs than its surrounding water body.

Occurrences of COCs in stream and sediment of different aquatic sample matrices were well documented in the literature (Ezemonye et al. 2008; Williams 2013; Olutona et al. 2014). Trend over time showed that various samples of aquatic environment are still contaminated with COCs such as polychlorinated biphenyls (PCBs) and hexachlorobenzenes (HBCs) (Agunloye 1984; Tongo 1985; Ogunlowo 1991; Nwankwoala and Osibanjo 1992; Idowu et al. 2013; Olutona et al. 2014). Moreover, Ogunfowokan et al. (2012) reported varying levels of COCs in sediment, water and benthic-dwelling organism samples of some aquatic systems where highest concentration was found in fish (benthic-dwelling organism) and least concentration in water sample. They ascribed processes of bioaccumulation and biomagnification to be responsible for this scenario as COCs are not readily metabolized, excreted and greatly lipid soluble, thereby biomagnify up the food chain (Ritter et al. 1995).

Despite the usage control legislation contained in the Pesticide Registration Regulation under Decree 15 of Federal Republic of Nigeria (FGN 1996), the production and use of agrochemicals including aldrin, binapacryl, captafol, chlordane, chlordimeform, DDT, dieldrin, dinoseb, ethylene dichloride, heptachlor, lindane, parathion, toxaphene, endrin, merix endosulphan, delta HCH and ethylene oxide etc. is yet to be abated as a result of illegal usage. Thus, the presence of pesticides in the environment emanates probably from the previous illegal use of these pesticide groups. The risk of the contamination of water column and bed sediment of aquatic environment is therefore of great concern due to the potential exposure of benthic-dwelling organisms such as fish and other edible aquatic organisms to the arrays of contaminants which could be transferred through different heterotrophic levels once in the food chain.

According to Zoumis et al. (2001), various interactions which exist among sediment, water, and aquatic organisms in a bionetwork could be regarded as major routes through which man is exposed to COCs by consuming contaminated edible aquatic foods along food chain in an environment. Crisp et al. (1998) also noted that a low dose of COCs in man might adversely affect his health because most of the COCs are easily absorbed through digestive system and bioaccumulate in the fatty tissue, adipose tissue, brain and female breast milk. Long term exposure to COCs has been reported to cause damage to liver and kidney (Siddharth et al. 2012), central nervous system (Costa et al. 2008), thyroid and bladder (Steenland et al. 2001). Higher concentrations of dieldrin, heptachlor and heptachlor epoxide have reported to block neurotransmitter in the central nervous system (Narahashi et al. 1992) including brain excitation, headache, confusion, muscle twitching, nausea and seizure (Lee et al. 2013).

Aiba stream provides water for domestic purposes and agricultural use. Physico-chemistry of this aquatic environment has been previously documented by Atobatele and Olutona (2013). As this area of study is predominantly surrounded by an irrigated farmland along with dumping of domestic waste and other anthropogenic activities, the use of pesticides is prevalent in this area for many years. The populace of Iwo is increasing on daily basis and majority of the inhabitant depends on this stream for their domestic and agricultural use (Olutona et al. 2012). Besides this, it is of equal importance to know the extent of pollution and impact of these pollutants on aquatic organism living in this stream, hence the need to determine the occurrence and distribution of COCs in Aiba stream. This investigation would provide more information on these organic contaminant concentrations on this aquatic ecosystem and on the possible health risk for local population depending on this stream for domestic use and consuming agricultural products emanated from this environment. At present, no data has been reported about the existence of COCs on Aiba stream.

## Methods

### Study area

**Fig. 1:**
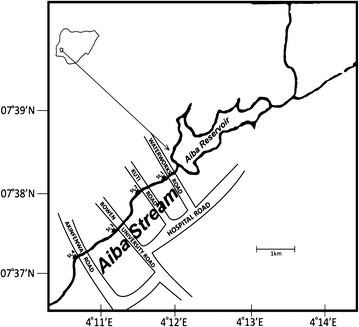
Map showing Aiba stream showing the sampling locations (adapted from Akindele and Liadi 2014)

Aiba stream in Iwo, Osun State, Nigeria (Fig. 1), is a unique aquatic habitat with its rich planktonic and zooplankton species. The stream provides water for domestic purposes and agricultural use. The area of study is predominantly surrounded by irrigated farmlands on which the use of pesticides is a common practice for many years. Anthropogenic activities on and around the stream is very high. These activities include car wash, domestic washing, spiritual bath, welding, filling station and fishing (Atobatele and Olutona 2013). Beside these, the attendant unsustainable indiscriminate dumping of domestic wastes into the stream has led to the poor water quality.

The study column on Aiba stream stretches from water column around the exit point or spillway where reservoir empties itself through water works area and drains through Kuti road, Bowen University road and Oke-Afo to the point of discharge into Oba River en-route Asejire dam (Fig. 1). Four sampling stations (SS) were identified namely: Water works (SS1) point of discharged water from the reservoir; Kuti area (SS2) runs through the residential area and there was car wash services and a local fish pond in this area; Oweyo area (SS3) is along a major road leading to private university associated with heavy traffic flow, residential and irrigation farmland; and Oke-Afo (SS4) downstream area characterised with dumping of refuse, welding and farmland area.

### Sediment sample collection

Field investigation took place at the peak of the wet season (September and October 2014) and the onset of dry season (December, 2014 and January, 2015). Samples of bottom sediment were collected in triplicates with grab sampler at a depth of 0 to 10 cm at each location. The geo-reference coordinates of the sampling points were obtained with a hand-held GPS Extrex Model. The collected samples were stored in clean aluminium foils and preserved in a refrigerator at about 4 °C prior analyses.

### Sample preparation

Sediment samples collected at each location were thwarted and made into composite representative samples. The samples were air-dried at ambient temperature on an inert surface in a ventilated cupboard to minimise cross contamination by atmospheric particulates. The dried sediment samples were then crushed and sieved through a 2 mm mesh pore size sieve to eliminate pebbles, large particle size materials and detritus substances.

### Apparatus and reagent used

Dichloromethane and n-hexane were purchased from GFS chemicals, (Inc Colombus, USA); acetone and hydrochloric acid from Park Scientific Ltd. (Northamptom, United Kingdom); Silical gel (silica gel 60, particle size 0.063–0.200 mm, 7–230 mesh) from Lab Tech Chemicals; and Sodium Sulphate from BDH, (England).

### Purification of solvents and chemicals

The solvents dichloromethane and n-hexane used for trace organics were triply distilled to obtain pure solvent that precluded all trace organic contaminants. Other materials such as glass wool, anhydrous sodium sulphate and silica gel were all heated in an oven at 105 °C for 1 h. The Whatman filter papers were oven dried to constant weights at 105 °C and cooled in a desiccator.

All glassware and bottles were previously soaked in 10 % nitric acid for 48 h, and then washed thoroughly with liquid detergent, water and thrice with distil water. The apparatus were then rinsed with acetone (95 %) then heated overnight in an oven at temperature of 105 °C prior to use.

### In-situ determination of physical and chemical parameters

The pH and electrical conductivity (EC) of the sediment samples were measured in situ using pH Testr water proof digital pH meter and EC Testr 2 dual range water proof digital EC meter. Water absorbency was determined according to the method described by Smith (1996). The sediment samples were oven-dried to a constant weight at 105 °C. The dried sediment samples were crushed using agate mortar using pestle. Thereafter, 3.0 g of sub-sample was weighed and soaked in water for 24 h. The excess water was drained and the sample re-weighed.

### Total organic carbon (TOC)

The TOC content of sediment samples were determined by weight loss or ignition method. Prior to the gravimetric determination, however, the sediment samples were dried to constant weight at 105 °C overnight to ensure a total removal of moisture. In each case, about 0.5 g of dried sediment samples was weighed into platinum crucible, ashed in a Vecsar muffle furnace model ECF 3 at 500 °C for 1 h. The furnace was allowed to cool, followed by weighing of the ignited sediments. The loss in weight of the residues gave the total organic carbon content. The percent organic carbon content of each sediment sample was calculated using the equation:

$${\text{Total}}\;{\text{organic}}\;{\text{carbon}}\; ( {\text{TOC)}}\;(\% ) = \frac{X - Y}{X} \times \frac{100}{1}$$

where X = initial weight of crucible and sediment before ignition (g); Y = final weight of crucible and sediment after ignition (g).

### Carbonate

A 0.5 g of dried sediment samples was placed in 25 mL distilled water. The solution was titrated with 0.01 M HCl using methyl red indicator. The carbonate content was calculated as CaCO_3_ total alkalinity.

### Extraction of COCs

Residues of COCs in the sediments samples were extracted by soxhlet extraction method described in the USEPA method 3540 (1996). Briefly, about 20 g of the <2 mm sieve size sediment sample were weighed into extraction thimble and placed in soxhlet extractors. The COCs were extracted by refluxing the samples with triply distilled dichloromethane at a temperature of about 40 °C, for about 10 h. The extracts were cooled to room temperature. Thereafter, each of the extracts was concentrated to about 2 mL under a stream of nitrogen gas (99.99 % purity).

### Extract cleanup

The COCs extracts were cleaned up using column chromatography (USEPA Method 3630C). Glass separating columns (15 cm × 1 cm i.d.) were packed with about 5 g activated silica gel. About 1 g of anhydrous sodium sulphate was placed at the top of each column to de-moisturize the sample extract. Each of the columns was pre-conditioned with 10 mL of n-hexane without the exposure of sodium sulphate layer to air. The samples were then loaded on the column and elution with 2 × 10 mL portion of dichloromethane. The eluate were collected, dried with anhydrous Na_2_SO_4_ and then concentrated to near dryness under stream nitrogen gas.

### Identification and quantification of COCs residues by gas chromatography

A gas chromatograph (GC–μECD HP GC 5890 series 11) coupled to ^63^Ni electron capture detector was used for the separation, identification and quantification of the COCS residues. The cleaned-up extracts were dried and re-dissolved in 1.0 cm^3^ analar grade n-hexane before injecting 1 μL of the purified extract into the injection port of the gas chromatograph.

## Instrument parameters

### Recovery study

Since no standard pesticide reference materials were available to us during the course of this study, recovery analysis was performed in order to ascertain the efficiency of the analytical procedures using standard methods. Recovery study was determined by spiking the previously analysed samples with pesticides standard. The recoveries of the COCs were determined by comparing the peak areas of the COCs after spiking with those obtained from the evaporated standard residue. Calculation of the % recoveries was as follows:

$$\%\,R = \frac{Conc\;of\;OCPs\;in\;spiked\;sample - Conc.\;of\;OCPs\;in\;unspiked\;sample \times 100}{Conc\;of\;added\;OCPs\;standard}$$

The limits of detection of COCs were determined by multiplying the standard deviation from the three replicates by three.

### Statistical analysis

The mean standard deviation for the organochlorine pesticide congeners from duplicate measurements was determined using the Statistical Package for the Social Science (SPSS) software, 15.0 for window evaluation version. Duncan’s multiple range tests was used to determine significant differences between means. The linear correlation coefficient of the COCS congeners was determined using the Pearson correlation coefficient. A principal component (PC) analysis was used to predict and identify possible sources of these contaminants by transforming the original set of data into a smaller set of principal components. Each component represents a new set of orthogonal variables that are a linear combination of the original variables. Each of the 18 COCS variables and six physicochemical parameters has a loading for each component, such that loadings reflect the correlation of the compound with that component. Loading equal or greater than 0.71 show that half of the variance in a variable is associated with that component, and this value was used to indicate high loadings of variable on a PC.

## Results and discussion

The result of the physio-chemical properties of the stream sediments are presented in Table 1. The results showed that sediment pH values were not significantly different from one another. The pH values ranged from 6.91 ± 0.00 at Kuti (SS2) to 7.70 ± 0.82 at Oke-Afo (SS4) with mean pH value of (7.12 ± 0.12). This showed that the bed sediment was generally neutral. However, sediments collected from Water Works (SS1) and Kuti water columns were slightly acidic, while those from Oweyo and Oke Afo water columns showed neutral downstream. The pH values were within the natural range (6.8–8.5) for freshwater ecosystem. Electrical conductivity ranged from 19 µS/cm at Kuti (SS2) and 39 µS/cm at Water works (SS1), with a mean value of 22.75 ± 16.69 µS/cm. Electrical conductivity of the sediment decreased downward along the course of the stream. Water absorbency (%) ranged from 27.17 % at Kuti (SS2) and 52.84 % at (Water works (SS1) with mean value of 41.88 ± 15.76 %. Wharfe (1977) observed that the finer clay/silt particles had a higher water retaining capacity (42–66 % water content) compared to more coarse sediment (28–49 % water content). The water retaining capacity can be important for burrowing invertebrates during periods of exposure (Olomukoro and Azubuike 2009). The water retention capacity observed in this study suggests that the benthic organisms that are bottom feeders would be very scanty in this running water ecosystem.

**Table 1:** Physico-chemical properties of stream sediment

Location	Cond. (µS/cm)	pH	Carbonate (mg/L)	TOC (%)	Temp (°C)	Water absorbency (%)
SS1	39.00 ± 33.94^a^	6.92 ± 1.04^a^	34.00 ± 8.49^a^	13.00 ± 4.24^a^	26 ± 0.00^a^	54.84 ± 19.57^a^
SS2	19.00 ± 5.66^a^	6.91 ± 0.00^a^	68.00 ± 59.40^a^	14.00 ± 0.00^a^	26 ± 1.41^a^	27.17 ± 0.71^a^
SS3	19.50 ± 3.51^a^	7.07 ± 0.22^a^	42.00 ± 25.46^a^	12.00 ± 2.83^a^	26 ± 1.41^a^	40.17 ± 23.33^a^
SS4	13.50 ± 2.12^a^	7.70 ± 0.82^a^	103.00 ± 46.57^a^	9.00 ± 1.41^a^	25.50 ± 0.71^a^	47.33 ± 8.48^a^
Mean ± SD	*22.75* ± 16.69	*7.12* ± 0.59	*61.75* ± 41.79	*12.00* ± 2.83	*25.88* ± 0.83	*41.88* ± 15.76

Mean with different letter of the alphabet for each column are significantly different at (p < 0.05) from each other

The organic carbon content ranged from 9 % at Oke-Afo (SS4) to 14 % at Kuti (SS2), with mean value of 12 ± 2.8 %. The organic content in bed sediment was low, which is consequent from large grain size. The study of this parameter is very important due to their implications in contaminant variability. Organic matter present in sediments constitutes a minor but important fraction of sediments (Karapanagioti et al. 2000) since it influences sediment structure (particles layout) which in turn determines the amount and strength of pollutants binding to the sediment (Hellar-Kihampa 2011). The organic carbon content and grain size of sediment influences chlorinated organic compounds; for instance pyrethroid bioavailability and toxicity (Holmes et al. 2008).

Carbonate values (mg/L) ranged from 34 mg/L at Water Works (SS1) to 103 mg/L at Ake-Afo (SS4) with mean value of 61.75 ± 41.79 mg/L. The carbonate contents in bed sediment increased downstream. The high carbonate content, indicate that they have a strong buffering capacity and it is very unlikely that, under natural condition, a significant acidification can be achieved.

Table 2 shows the results of retention time, percentage recovery and limit of detection (LOD) of COCs in sediment samples. The retention time (min) ranges from α-BHC (9.66 ± 0.03) to Trans-Pemerthrin (21.60 ± 0.14). The percentage recovery of the eighteen organochlorine compounds ranges from 82.68 to 95.10 % while the LOD values range from 0.03 to 0.61 ppm. These indicated that the separation efficiency of the method for µGC–ECD identification and quantification method was efficient. The standard deviations also show that the reproducibility of the results is satisfactory.

**Table 2:** Retention time, % recoveries and LOD values

Pesticide	Retention time	% Recovery	LOD value	Pesticide	Retention time	% Recovery	LOD value
α-BHC	9.66 ± 0.03	84.56 ± 4.58	0.09	Dieldrin	16.02 ± 0.07	83.88 ± 2.38	0.21
β-BHC	10.15 ± 0.09	95.10 ± 6.70	0.27	Endrin	16.43 ± 0.09	82.68 ± 6.21	0.27
Lindane	10.47 ± 0.01	82.69 ± 5.25	0.03	Endosulfan II	16.81 ± 0.08	86.78 ± 3.99	0.24
δ-BHC	10.82 ± 0.17	90.34 ± 4.32	0.51	ppDDD	17.16 ± 0.14	89.78 ± 2.23	0.42
Chlorothalonil	11.03 ± 0.07	93.55 ± 6.85	0.21	Endosulfan sulfate	17.88 ± 0.05	89.74 ± 5.12	0.15
Heptachlor	11.88 ± 0.01	89.78 ± 8.70	0.03	pp DDT	18.27 ± 0.08	92.75 ± 4.44	0.24
Aldrin	12.73 ± 0.04	90.56 ± 4.53	0.12	Lambda Cyhalothrine	20.76 ± 0.10	85.79 ± 5.08	0.30
Heptachlor epoxide (B)	14.01 ± 0.21	89.43 ± 7.89	0.61	Cis-Permethrin	21.24 ± 0.04	86.65 ± 3.65	0.12
Endo Sulfan1	15.27 ± 0.08	84.23 ± 4.80	0.24	Trans Permethrin	21.60 ± 0.14	90.32 ± 4.27	0.42

The distribution of COCs (µg/g) in sediment samples in all the four locations are presented in Tables 3 and 4. A total of eighteen compounds were detected. Nearly all the compounds were detected in each of these locations with the exception of Heptachlor epoxide (B) which was not detected at Kuti (SS2); and endosulfan sulphate and Cis-Permerthrim that were not detected at Kuti (SS2) and Oweyo (SS3) water column. The specific COCs compounds detected in the samples and their concentrations differ markedly, indicating wide spread contamination by these compounds in the stream. The mean concentration of COCs (µg/g) in sediment samples were as follows: α-BHC (0.23 ± 0.20), β-BHC (0.29 ± 0.29), δ-BHC (0.30 ± 0.24), Lindane (6.79 ± 8.60), Chlorothalonil (0.18 ± 0.08), Heptachlor (0.26 ± 0.33), Heptaclor epoxide (0.30 ± 0.40), Aldrin (0.44 ± 0.51), Dieldrin (0.04 ± 0.03), Endrin (0.05 ± 0.04), Endosulfan I (0.35 ± 0.80), Endosulfan II (0.28 ± 0.28), Endosulfan sulphate (0.03 ± 0.05), pp-DDD (0.08 ± 0.13), ppDDT (4.69 ± 8.49), λ-Cyhalothrine (33.85 ± 41.69), Cis-Permethrin (0.77 ± 1.29) and Trans-Permethrin (375.70 ± 689.41).

**Table 3:** Levels (µg/g) of organochlorine in bed sediment of Aiba stream

Location	α-BHC	β-BHC	δ-BHC	Lindane	Chlorothalonil	Heptachlor	Heptachlor epoxide(B)	α-Endo sulfan	β-Endo sulfan	Endosulfan sulfate
*(a)*										
SS1	0.11 ± 0.05^a^	0.11 ± 0.09^a^	0.09 ± 0.00^a^	1.54 ± 1.74^a^	0.16 ± 0.00^a^	0.10 ± 0.06^a^	0.43 ± 0.61^a^	0.05 ± 0.00^a^	0.06 ± 0.01^a^	0.06 ± 0.08^a^
SS2	0.17 ± 0.01^a^	0.24 ± 0.07^a,b^	0.24 ± 0.22^a,b^	1.37 ± 1.87^a^	0.09 ± 0.13^a^	0.08 ± 0.09^a^	Nd	1.16 ± 1.61^a^	0.45 ± 0.24^a^	Nd
SS3	0.14 ± 0.06^a^	0.10 ± 0.09^a^	0.27 ± 0.25^a,b^	13.81 ± 16.44^a^	0.20 ± 0.01^a^	0.23 ± 0.27^a^	0.42 ± 0.59^a^	0.22 ± 0.27^a^	0.49 ± 0.45^a^	Nd
SS4	0.50 ± 0.29^a^	0.69 ± 0.34^b^	0.61 ± 0.00^b^	10.45 ± 1.11^a^	0.25 ± 0.03^a^	0.62 ± 0.55^a^	0.36 ± 0.42^a^	0.03 ± 0.03^a^	0.14 ± 0.18^a^	0.06 ± 0.08^a^
Mean ± SD	0.23 ± 0.20	0.29 ± 0.29	0.30 ± 0.24	6.79 ± 8.60	0.18 ± 0.08	0.26 ± 0.33	0.30 ± 0.40	0.35 ± 0.80	0.28 ± 0.28	0.03 ± 0.05

Location	Aldrin	Dieldrin	Endrin	pp-DDD	pp-DDT	Lambda cyhalothrine	Cis-permethrin	Trans permethrin
*(b)*								
SS1	0.14 ± 0.12^a^	0.05 ± 0.00^a^	0.04 ± 0.01^a^	0.01 ± 0.01^a^	1.04 ± 1.23^a^	4.57 ± 6.22^a^	1.79 ± 1.83^a^	54.73 ± 74.17^a^
SS2	0.29 ± 0.28^a^	0.07 ± 0.02^a^	0.03 ± 0.04^a^	0.14 ± 0.20^a^	12.58 ± 16.74^a^	77.75 ± 66.18^a^	Nd	102.21 ± 144.55^a^
SS3	0.16 ± 0.17^a^	0.24 ± 0.03^a^	0.04 ± 0.06^a^	0.14 ± 0.19^a^	4.74 ± 5.88^a^	38.52 ± 31.37^a^	Nd	359.43 ± 508.32^a^
SS4	1.19 ± 0.42^b^	0.03 ± 0.03^a^	0.08 ± 0.01^a^	0.02 ± 0.02^a^	0.40 ± 0.26^a^	14.56 ± 20.17^a^	1.31 ± 1.85^a^	986.41 ± 1392.90^a^
Mean ± SD	0.44 ± 0.51	0.04 ± 0.03	0.05 ± 0.04	0.08 ± 0.13	4.69 ± 8.49	33.85 ± 41.69	0.77 ± 1.29	375.70 ± 689.41

Mean with different letter of the alphabet for each column are significantly different at (p < 0.05) from each other

**Table 4:** Two tailed correlation coefficient matrix for individual COCs

	αBHC	βBHC	Lind	δBHC	Chlor	Hept	Aldrin	HepEpox	αEndo	Dield	Endrin	βEndo	DDD	EndoSul	DDT	λCyhal	CisP	TransP
αBHC	1																	
βBHC	0.98**	1																
Lind	0.28	0.22	1															
δBHC	0.72*	0.78*	0.59	1														
Chlor	0.49	0.51	0.377	0.681	1													
Hept	0.234	0.315	0.54	0.72*	0.45	1												
Aldrin	0.91**	0.89**	0.11	0.62	0.36	0.35	1											
HepEpox	0.12	0.02	−0.30	−0.20	0.29	−0.27	0.26	1										
αEndo	−0.15	−0.19	−0.22	−0.42	−0.89**	−0.32	0.01	−0.22	1									
Dield	−0.36	−0.27	−0.08	−0.19	−0.72*	−0.04	−0.32	−0.69	0.53	1								
Endrin	0.49	0.56	0.71	0.88**	0.69	0.71*	0.29	−0.37	−0.62	−0.06	1							
βEndo	−0.26	−0.28	0.61	0.07	−0.41	0.15	−0.33	−0.59	0.46	0.49	0.12	1						
DDD	−0.19	−0.29	−0.32	−0.51	−0.59	−0.43	0.02	0.24	0.78*	−0.07	−0.81*	0.18	1					
EndoSul	0.52	0.46	−0.12	0.15	0.31	−0.14	0.53	0.69	−0.27	−0.33	0.07	−0.55	−0.24	1				
DDT	−0.21	−0.27	−0.28	−0.51	−0.87**	−0.39	−0.04	−0.15	0.98**	0.41	−0.71*	0.40	0.87**	−0.49	1			
λCyhal	−0.27	−0.29	−0.24	−0.39	−0.80*	−0.25	−0.09	−0.24	0.94**	0.42	−0.63	−0.48	0.85**	−0.49	0.96**	1		
CisP	0.49	0.46	−0.10	0.02	0.28	−0.26	0.31	0.07	−0.30	−0.26	0.17	−0.57	−0.32	0.39	0.29	−0.48	1	
TransP	0.01	0.09	0.09	0.39	0.29	0.80*	0.27	0.09	−0.17	−0.11	0.27	−0.10	−0.04	−0.33	−0.15	0.03	−0.32	1

Chlorothalonil, λ-Cyhalothrine, Cis and trans-Permethrin were not detected in Aiba Reservoir in an earlier study of COCs conducted by Olutona et al., (2014). Literature concerning their presence especially in bed sediments of Nigerian Rivers are scarce and scanty. Recently Sikoki et al. (2014) reported that, Chlorothalonil; (a non-systemic fungicide) and Pyrethroid pesticides are widely used in offices and residential buildings, agricultural fields, animal and greenhouses. Permethrin on the other hand is used in skin lotion and shampoos as medical treatment of lice and scabies. The higher concentrations of λ-Cyhalothrine (33.85 µg/g), Cis-Permethrin (0.77 µg/g) and *Tans*-Permethrin (375.70 µg/g) detected in this study, showed the presence of not only agrochemicals products, but the inclusion of personal care products arising from domestic wastes dumped into the stream.

Pyrethroids have been promoted, many places around the globe, especially in Nigeria. They have been used for the treatment of bed nets, as an alternative to indoor residual spray of insecticides to prevent malaria spread (Barlow et al. 2001). Global focus on the use of synthetic pyrethroids as an alternative means of rolling back malaria, and the reduction on the reliance on DDT may be the reason for the elevation of the concentrations of pyrethroids compounds recorded in the bed sediment of Aiba stream. Although residues of DDE were not found in all the samples, the mean concentration of DDT was about 60 times higher than that of DDD. Both DDD and DDE are degradation product of DDT (Yanez et al. 2002). The low concentration of pp-DDD, and non-detection of pp-DDE in Aiba stream sediment indicated that, lower degradation of DDT has occurred. This also suggests the likelihood of fresh DDT application, and/or old accumulation of this compound. When switching from one chemical compound to another and perhaps especially when moving away from DDT, it is inescapable that residue of the former will remain for extended period (Yanez et al. 2002). The higher concentration of pp-DDT (4.69 µg/g) found in the sediment of the investigated aquatic environment catchment may be linked with the frequent use or application of agrochemicals, and urban runoff of water abused by dumping of refuse along the water course.

The three isomers of benzene hexachloride (BHC) detected in all locations evaluated in this study were heterogeneously distributed. The trend for all the isomers, revealed an increasing concentrations of α, β and δ BHC isomers downstream along the course of the stream. The γ-isomer of hexa-chlorocyclohexane (HCH) or benzene hexachloride (BHC), lindane had mean concentration of 6.79 ± 8.60 µg/g with higher concentrations observed down the stream, especially at Oweyo (SS3) and Oke-Afo (SS4) water column catchment. This may be due to the use of agricultural pesticides along the course of the river downstream and perhaps sedimentation.

The levels of aldrin obtained in this study ranged from (0.14 µg/g) at Water Works (SS1) to (1.19 µg/g) at Oke-Afo (SS4) with mean value of (0.44 ± 0.51 µg/g), while dieldrin ranged from) 0.03 µg/g) at Oke-Afo (SS4) to (0.24 µg/g) at Oweyo (SS3) with mean value of (0.04 ± 0.03 µg/g). In the environment, aldrin gets converted to Dieldrin, which is more stable. In this study, the concentrations of aldrin and dieldrin were in ratio 10:1, which suggested high persistence of this chemical in the environment. Nollet (2000) noticed that aldrin strongly adsorbs to sediment particles and remains intact for longer period in the aquatic environment. Furthermore, sunlight and bacteria alter aldrin to dieldrin, degrading them slowly in sediments (ATSDR 2002). Low levels of anthropogenic inputs accounted for high disparity in the concentrations of aldrin and dieldrin detected in this study compared with similar studies carried out by Darko et al. (2008) and Kuranchie-Mensah et al. (2012) in Ghana, who reported the concentrations of aldrin and dieldrin in Lake Bosomtwi in Ashanti region as 0.65 and 0.072 µg/kg; 10.98 and 0.313 µg/kg in Weija; and 6.348 and 0.250 µg/kg in Nsawam region of Densu river basin, respectively.

Endrin was detected in all the sampling stations in a similar trend. Endrin once in water strongly adsorbed to sediment thereby removing the chemical from water, and concentrate in sediment (ATSDR 1996). Endrin undergoes photo degradation to endrin aldehyde and endrin ketone (Nollet 2000). The absence of endrin aldehyde and endrin ketone the degradation product of endrin in this study gave indication of fresh input of this compound.

Due to the rapid conversion process of synthetic organochlorine heptachlor to heptachlor epoxide, a major persistent degradation product (heptachlor epoxide) is abundant in the environment. As a result of its hydrophobicity and affinity for organic materials, heptachlor epoxide tends to be associated with particulate matter in aquatic environment, hence their accumulation in the bed sediment (Environment Canada 1999). In this study, the mean concentration of heptachlor epoxide was slightly higher than its parent compound i.e. heptachlor. Adverse biological effects of heptachlor epoxide in bed sediment of fresh water include decreased diversity, reduced abundance, increased mortality and behavioural changes in benthic organisms (Environment Canada 1999).

The observed mean values (µg/g) of two stereoisomers α, β and endosulfan sulphate were 0.35 ± 0.80, 0.28 ± 0.28 and 0.03 ± 0.05, respectively. These stereoisomers, α- and β-with different physical properties are associated with sediment in fresh water ecosystem, while endosulfan sulphate is commonly found in sediments as the product of biotic and abiotic degradation. All three isomers have been reported to be very toxic as parent compound to aquatic habitat especially fish (Pandey et al. 2011). The results showed that the concentration of α- was slightly higher than β, while endosulfan sulphate was low, perhaps as a result of slow formation during the process of degradation, vis-a-viz pH-induced hydrolysis. In slightly alkaline water, hydrolysis is likely to be rapid, while low temperatures significantly reduce the rate of hydrolysis. The neutral pH value of sediment in this study might be a contributing factor to slow degradation of endosulfan to their sulphate. The low sediment temperature obtained in this study might also contribute to low degradation process of isomers of endosulfan to their sulphate. Furthermore, the higher mean values of α-endosulfan isomer might be due to fact that the manufactured technical endosulfan normally contain 67 % α-endosulfan by weight of total endosulfan content while β-endosulfan constitute only 32 % (WHO 1997); and α-endosulfan is thermally stable while its β isomer is unstable and is converted to the α-isomer in the environment (Rice et al. 1997; Hapeman et al. 1997). Idowu et al. (2013) also observed higher content of α-endosulfan than its β-isomer in river water and sediment of cocoa producing area of Ondo, Nigeria.

Some recent studies on the levels of COCs in the sediment in Nigerian rivers reported are as follows: Northern Nigeria, Okeniyia et al. (2009) showed that COCs ranged from 0.0008 to 0.285 ppb. In south eastern Nigeria studies of COCs in sediment of Warri River of the Niger Delta, Nigeria ranged from <LOD −12.60 µg/g (Ezemonye et al. 2008); 0.06–11.9 µg/g dry weight Ezemonye et al. (2010); the concentrations of target 21 COCs in surface sediment of selected rivers, canals and streams of the Niger Delta ranged from 20 to 313 ng/g with a mean value of 102 ng/g (Olatunbosun et al. 2011). In south western Nigeria studies of COCs in rivers near cocoa producing areas where organochlorine pesticides were predominantly used have been assessed and reported by various authors, Okoya et al. (2013) reported cis-chlordane (0.03–6.99 µg/g), α-endosulfan (0.03–6.99 µg/g), pp-DDE (0.08–19.04 µg/g) and dieldrin (0.01–7.62 µg/g); (Nd-127.14 mg/kg) Idowu et al. (2013); (Nd-691.7 ± 5.8 ng/g) Williams (2013); and (0.0455–5.885 µg/kg) Ibigbami (2013). Findings in this study significantly differ greatly from findings of COCs concentrations in other river systems recently studied from similar agro-ecological zones in Nigeria. This could be attributed to the use of agrochemical products, dumping of domestic sewage into the stream and the water course running through residential areas. Brigham et al. (1996) in their studies of trace and organic chemicals in stream-bottom sediments and fish tissues, of Red River, Minnesota, North and South Dakota reported that most of the organochlorine analyzed were not detected in all samples, the DDT metabolites were frequently detected which ranged between 8.9 and 23 ng/g. Philips et al. (2010) in their studies that aimed to identify mixtures and modified concentrations addition approach to estimate the potential toxicity of COCs and PCBs at 85 streams across the United State reported that the principal components (PC) analysis identified five PCs accounted for 77 % of the total variance in 14 organochlorine compounds in the original data sets. The PC analysis grouped compounds that have similar chemical structure, common origin and similar relation of concentrations to land use. Hellar-Kihampa (2011) studied pesticides residues in four rivers in Tanzania reported that COCS concentrations ranged from trace (endrin) to 120 ng/l (p,p′ –DDD) in water, and from trace (aldrin) to 132 ng/g-dry weight (p,p′ –DDD) in sediments, and were higher during the dry season. Concentrations of total dichlorodiphenyltrichloroethanes (DDTs) and hexachlorocyclohexanes (HCHs) in surface sediment of Bohai Sea, China ranged widely from 0.24 to 5.67 ng/g (mean 1.36 ± 0.93 ng/g) and 0.16 to 3.17 ng/g (mean 0.83 ± 0.57 ng/g), respectively (Hu et al. 2009).

### Correlation analysis

Correlation analysis was performed for COCs by using two tailed Pearson correlation coefficient to determine relationships between individual compounds. Detailed analysis of the data set revealed that there were some significant positive correlations between some components at either p < 0.01 or p < 0.05 levels. Similarly, few compounds showed negative correlation both at p < 0.01 and p < 0.05 level. α, β-BHC was found to be positively correlated with each other as well as with its δ isomers and with aldrin. δ-BHC was positively correlated with heptachlor and endrin both at p < 0.05 and p < 0.01 levels, respectively while heptachlor was positively correlated with endrin at p < 0.05 level. α-Endosulfan with DDD, DDT and λ-Cyhalothrine both at p < 0.05 and p < 0.01 levels. DDD was found to be positively correlated with DDT and both isomers were positively correlated with λ-Cyhalothrine at p < 0.01 and p < 0.05 levels. Chlorothalonil was found to be negatively correlated with DDT, α-endosulfan, λ-Cyhalothrine and dieldrin at p < 0.01 and p < 0.05 levels. Endrin was also found to be negatively correlated with both DDD and DDT at p < 0.05 level.

Positive correlations that exhibited among these COCS compounds were an indication that these compounds have a common source of origin and similar environmental behaviour. Moreover, this can also be attributed to the fact that all COCs are related derivatives, and that they are commonly used in agriculture and vector control programmes. Further, since these compounds bind tightly to soil and sediments, this explains their presence even long time after discharge (Pandey et al. 2011). Negative correlation might be attributed to different chemical structures among these compounds.

### Principal component analysis

Distribution pattern of the observed chlorinated organic compounds in this study was investigated using principal component analysis (PCA). This analysis reduced the observed variables down to their principal components while maximizing the variance accounted for in the variables by the components.

**Fig. 2:**
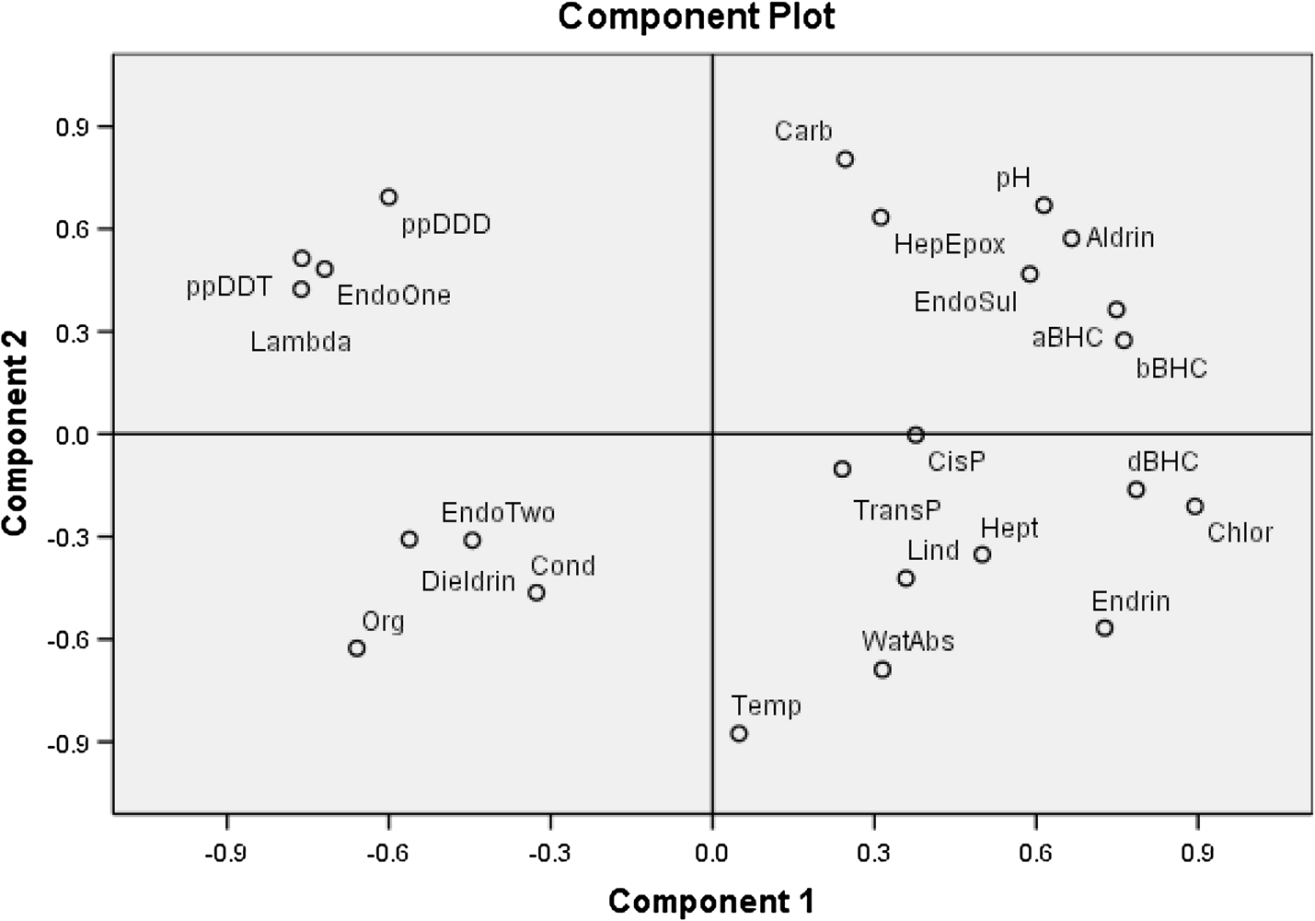
Loadings for the first two PC, from PA of OCP and Physicochemical parameters in stream bottom sediment. *Carb* Carbonate, *HepEpox* Hepthachlor Epoxide (B), *EndoSul* Endosulfan Suphate, *CisP* Cis-Permethrin, *TransP* Trans-Permethrin, *Lind* Lindane, *Hept* Heptachlor, *Chlor* Chlorothanil, *EndoTwo* β-EndoSulfan, *Cond* Conductivity, *WatAbs* Water absorbency, *Temp* Temperature, *Org* Organic carbon, *EndoOne* α-EndoSulfan, *Lambda* Lambda Cyhalothrine

The PCA yielded loading of the variables (18 COCs and six physiochemical parameters) onto six components at eigen value (the standardized variance associate with a particular component) greater than 1.0 and this was summarized (Table 5). The six components accounted for a total of 97 % of the total variance in the 24 compounds. All the derivatives of hexachlorobenzene, Chlorothanolil and Endrin had high loadings (>0.7) in the first component. Carbonate had a high loading of 0.80 followed by pp-DDT with moderate loading of 0.69 in the second component. A plot of PC2 versus PC1 (Fig. 2) shows useful groupings of COCs and physico chemical parameters determined. Due to the fact that COCs are hydrophobic and non-ionizing organic compounds, the percentage of water absorbency and soil pH might have little association with the COCs. Meanwhile, adsorption of hydrophobic organic pollutants such as COCs is strongly dependent on the soil organic matter content; this might be the like reason for the association between organic carbon and COCS such as dieldrin and β Endosulfan. Soil organic matter may vary from soil to soil in its polarity, elemental composition, aromaticity, condensation, and degree of diagenetic evolution from a loose polymer to condensed coal-like structures (Karapanagioti et al. 2000). Therefore land variations, such as type and age of soil organic matter may affect sorption of non-ionic pesticides.

**Table 5:** Component loadings for OCPs and physicochemical parameters of stream sediment data

	Component					
	1	2	3	4	5	6
aBHC	0.749	0.364	0.277	0.445	0.093	0.044
bBHC	0.763	0.274	0.312	0.425	0.054	−0.114
Lind	0.359	−0.422	0.520	0.002	0.259	0.553
dBHC	0.785	−0.162	0.559	0.000	0.065	−0.045
Chlor	0.894	−0.211	−0.138	−0.198	−0.122	0.208
Hept	0.500	−0.352	0.607	−0.232	−0.345	−0.137
Aldrin	0.665	0.572	0.357	0.280	−0.125	−0.090
HepEpox	0.312	0.634	−0.532	−0.390	0.009	0.155
EndoOne	−0.719	0.483	0.422	0.236	0.072	−0.038
Dieldrin	−0.562	−0.308	0.361	0.250	0.223	−0.544
Endrin	0.727	−0.567	0.358	0.094	0.109	−0.004
EndoTwo	−0.445	−0.311	0.666	−0.082	0.388	0.315
ppDDD	−0.600	0.693	0.149	−0.086	−0.174	0.309
EndoSul	0.588	0.468	−0.373	0.065	0.386	−0.199
ppDDT	−0.761	0.514	0.336	0.193	−0.019	0.063
Lambda	−0.762	0.423	0.468	0.025	−0.098	0.027
CisP	0.377	−0.003	−0.477	0.783	−0.112	0.072
TransP	0.240	−0.102	0.429	−0.355	−0.771	−0.146
WatAbs	0.315	−0.689	−0.037	0.449	−0.183	0.158
Carb	0.246	0.804	0.381	0.382	−0.017	−0.004
Temp	0.049	−0.876	0.020	−0.264	0.330	−0.177
Cond	−0.326	−0.464	−0.396	0.637	−0.304	0.139
pH	0.614	0.669	−0.014	−0.221	0.337	−0.032
Org	−0.659	−0.626	−0.023	0.358	0.115	0.001

## Conclusion

The study revealed the presence of eighteen organochlorine pesticides compounds at various concentrations in sediment samples of Aiba stream. The high levels of these pesticides were not surprising since these compounds were readily adsorbed to sediment particles. The pesticides detected on Aiba stream may have originated from agricultural activities taking place along the bank of the stream and subsequent runoff of COCs into the water body coupled with indiscriminate dumping of domestic waste by the residents living along the bank of the stream.

## References

[CR5] Agency for Toxic Substancea and Disease Registry (ATSDR) (1996) Toxicological profile for hexachlorobenzene. 1600 clifton road NE, E-29, Atlanta, georgia 37307367

[CR1] Agency for Toxic Substances and Disease Registry (ATSDR) (2002). Toxicological profile for aldrin and dieldrin.

[CR2] Agunloye TO (1984) A survey of chlorinated hydrocarbons in Rivers of Southern Nigeria. Ph.D. thesis, Department of Chemistry, University of Ibadan, Nigeria. pp 254

[CR3] Akindele EO, Liadi AA (2014). Diversity and response of benthic macro invertebrates to natural and induced environmental stresses in Aiba Stream Iwo, Southwestern Nigeria. West Afr J Pure Appl Ecol.

[CR4] Aly Salem DMS, Khaled A, Nemr El (2013). Asssessment of pesticides and polychlorineted biphenyls (PCBs) in sediment of the Egyptian Mediterranean Coast. Egypt J Aquat Res.

[CR6] Atobatele EO, Olutona GO (2013). Spato-seasonal physico-chemistry of Aiba Stream, Iwo, Nigeria. Afr J Biotechnol.

[CR7] Barlow SM, Sullivan FM, Lines J (2001). Risk assessment of the use of deltamethrin on bednets for the prevention of malaria. Food Chem Toxicol.

[CR8] Basheer C, Lee HK, Obbard JP (2002). Determination of organochlorine pesticides in sea water using liquid-phase hollow fibre membrane mocro extraction and gas chromatography–mass spectrometry. J Chromatogr A.

[CR9] Brasher AM, Anthony SS (2000). Occurrence of organochlorine pesticides in stream bed sediment and fish from selected streams on the island of Oahu, Hawaii, 1998. Manoa.

[CR10] Brigham ME, Goldstein RM, Tornes LH (1996). Trace elements and organic chemicals in stream-bottom sediments and fish tissues, Red River of the North Basin, Minnesota, North Dakota, and South Dakota, 1992–95.

[CR11] Costa LG, Giordano G, Giizzetti M, Vitalone A (2008). Neurotoxicity of pesticides: a brief review. Front Biosci.

[CR12] Crisp TM, Clegg ED, Cooper RL, Wood WP, Andersen DG, Baetcke KP, Hoffmann JL, Morr MS, Rodier DJ, Schaeffer JE, Touart LW, Zeeman MG, Patel YM, Clegg ED (1998). Environmental endocrine disruption: an effects assessment and analysis. Environ Health Perspect.

[CR13] Darko G, Akoto O, Opong C (2008). Persistent organochlorine pesticide residue in fish, sediment and water from Lake Bosomtwi, Ghana. Chemosphere.

[CR14] Environment Canada (1999) Canadian Environmental Protection Act. http://www.ec.gc.ca/CEPARRegistry/the_act/. Accessed 23 Sept 2011

[CR15] Ezemonye LK, Ikpesu TO, Tongo I (2008). Distribution of lindane in water, sediment and fish from Warri Rivers of the Niger Delta, Nigeria. Arhiv za Hijigenu Radai Toksikologiju.

[CR16] Ezemonye LK, Ikpesu TO, Tongo I (2010). Distribution of endosulfan in water, sediment and fish from Warri River, Niger Delta, Nigeria. Afr J Ecol.

[CR17] Federal Republic of Nigeria Official Gazette (FGN) (1996) Federal Government Press, Lagos, Nigeria, vol 83, pp 303–307

[CR18] Gilliom RJ (2007). Pesticides in US streams and groundwater. Environ Sci Technol.

[CR19] Hakanson L, Burton GA (1992). Sediment variability. Sediment Toxicity Assessment.

[CR20] Hapeman CJ, Schdimt WF, Rice CP (1997) Structural and thermodynamics considerations in the isomeric conversion of endosulfan. In: 213th National meeting of the American Chemical Society, San Franscico, Califonia, USA, April 13–17

[CR21] Hellar-Kihampa H (2011). Pesticide residue in four rivers running through an intensive agricultural area, Kilimanjaro, Tanzania. J Appl Sci Environ Manag.

[CR22] Holmes RW, Anderson BS, Philips BM, Hunt JW, Crane DB, Mekebri A, Connor V (2008). State wide investigation of the role of pyrethroid pesticides in sediment toxicity in Califonia’s Urban Waterways. Environ Sci Technol.

[CR23] Hu L, Zhang G, Zheng B, Qin Y, Lin T, Guo Z (2009). Occurrence and distribution of organochlorine pesticides (COCs) in surface sediments of the Bohai Sea, China. Chemosphere.

[CR24] Ibigbami OA (2013). Levels of organochlorine pesticides (COCs) residue in selected cocoa farms in Ilawe-Ekiti, Ekiti State, Nigeria. Anal Open J Res.

[CR25] Idowu GA, Aiyesanmi AF, Owolabi BJ (2013). Organochlorine pesticide residue levels in river water and sediment from cocoa-producing areas of Ondo State central senatorial district, Nigeria. J Environ Chem Ecotoxicol.

[CR26] Karapanagioti HK, Kleineidam S, Sabatini DA, Grathwohi P, Ligouis B (2000). Impacts of heterogeneous organic matter on phenanthrene sorption: equilibrium and kinetic studies with aquifer material. Environ Sci Technol.

[CR27] Kuranchie-Mensah H, Atiemo SM, Naa-Dedei Palm LM, Blankson-Arthur S, Tutu AO, Fosu P (2012). Determination of organochlorine pesticide residue in sediment and water from the Densu River Basin, Ghana. Chemosphere.

[CR28] Lee SH, Ra JS, Choi JW, Yim BJ, Jung MS, Kim SD (2013). Human health risks associated with dietary exposure to persistent organic pollutants (POPs) in river water in Korea. Sci Total Environ.

[CR29] Narahashi T, Frey JM, Ginsburg KS, Roy ML (1992). Sodium and GABA-activated channels as the targets of pyrethroids and cyclodienes. Toxicol Lett.

[CR30] Nollet LML (2000). Handbook of water analysis food science and technology.

[CR31] Nwankwoala AU, Osibanjo O (1992). Baseline levels of selected organochlorine pesticide residues in surface waters in Ibadan (Nigeria) by electron capture gas chromatography. Sci Total Environ.

[CR32] Ogunfowokan AO, Oyekunle JAO, Torto N, Akanni MS (2012). A study in persistence organochlorine pestiside residues in fish tissues and water from an agricultural fish pond. Emir J Food Agric.

[CR33] Ogunlowo SO (1991) Priority chemical pollutants in some rivers along the cocoa growing area of Ondo State. Ph.D. dissertation Dept. of Chemistry, University of Ibadan, Ibadan, Nigeria

[CR34] Okeniyia SO, Egwaikhideb PA, Akporhonorc EE, Obazed IE (2009). Distribution of organochlorine and polychlorinated residues in water bodies of some rivers in Northern Nigeria. Electron J Environ Agric Food Chem.

[CR35] Okoya AA, Ogunfowokan AO, Asubiojo OI, Torto N (2013) Organochlorine pesticide residues in sediments and water from producing areas of Ondo State, southwestern Nigeria. Soil Sci. doi:10.1155/2013/131647

[CR36] Olatunbosun S, Sojinu S, Sonibare OO, Ekundayo O, Zeng EY (2011). Occurrence of organochlorine pesticides (COCs) in surface sediments of the Niger Delta, Nigeria. J Appl Sci Res.

[CR37] Olomukoro JO, Azubuike CN (2009). Heavy metals and macroinvertebrates communities in bottom sediment of Ekpan Creek, Warri, Nigeria. Jordan J Biol Sci.

[CR38] Olutona GO, Akintunde EA, Otolorin JA, Ajisekola SA (2012). Physico-chemical quality assessment of shallow well-waters in Iwo, Southwestern Nigeria. J Environ Sci Water Res.

[CR39] Olutona GO, Ayano SA, Obayomi-Davies O (2014). Organochlorine pesticide in water and bottom sediment from Aiba reservoir (Southwestern Nigeria). Chem Ecol.

[CR40] Pandey P, Khillare PS, Kumar K (2011). Assessment of organochlorine pesticides residues in the surface sediments of river Yamuna in Delhi India. J Environ Prot.

[CR41] Philips PJ, Nowell LH, Gillion RJ, Nakagaki N (2010). Composition, distribution, and potential toxicity of organochlorine mixtures in bed sediments of Streams. Sci Total Environ.

[CR42] Rice CP, Hapeman CJ, Chemyalk SM (1997) Experimental evidence of the interconversion of endosulfan isomers. In: 213th National meeting of the American Chemical Society, San Franscico, Califonia, USA, April 13–17

[CR43] Ritter L, Solomon KR, Forget J (1995) Persistent organic pollutants. Retrieved February 16, 2004, from http://www.chem.unep.ch/pops/ritter/en/ritteren.pdf

[CR44] Siddharth M, Datta SK, Bansal S, Mustafa M, Banerjee BD, Kalra OP, Tripathi AK (2012). Study on organochlorine pesticide levels in chronic kidney disease patients: association with estimated glomerular filtration rate and oxidative stress. J Biochem and Mol Toxicol.

[CR45] Sikoki FD, Lelei KE, Gbarakon TN, Vincent-Akpu IF, Brambaifa B (2014). Assessment of organochlorine pesticides (COCs) residues in the Bonny Estuary in the Niger Delta Area of Nigeria. Int J Geol Earth Environ Sci.

[CR46] Smith PT (1996). Physical and chemical characteristics of sediments from prawn farms and mangrove habitats on the Clarence River, Australia. Aquaculture.

[CR47] Ssebugere P, Kiremirea BT, Henkelmannb B, Bernhöft S, Kasozia GN, Wasswaa J, Schramm KW (2013). PCDD/Fs and dioxin-likePCBs in fishs pecies from Lake Victoria, EastAfrica. Chemosphere.

[CR48] Ssebugere P, Sillanpää M, Kiremire BT, Kasozi GN, Wang P, Sojinu SO, Otieno PO, Zhu N, Zhu C, Zhang H, Shang H, Ren D, Li Y, Zhang Q, Jiang G (2014). Polychlorinated biphenylsand hexachlorocyclohexanes in sediments and fish species from the Napoleon Gulf of Lake Victoria, Uganda. Sci Total Environ.

[CR50] Steenland K, Mannetje A, Boffetta P, Stayner L, Attfield M, Chen J, Checkoway H (2001). Pooled exposure–response analyses and risk assessment for lung cancer in 10 cohorts of silica-exposed workers: an IARC multicentre study. Cancer Causes Control.

[CR51] Tongo AA (1985) Baseline study of levels of organochlorine pesticides in Nigerian rivers and their sediments. M. Sc Thesis, Department of Chemistry, University of Ibadan, Nigeria

[CR52] Wharfe JR (1977). An ecological survey of benthic invertebrates macrofauna of the lower Medway Estuary, Kent. J Anim Ecol.

[CR53] WHO (World Health Organization (1997) Chemistry and specifications of pesticides. Thirteenth reports on the WHO expert committee on vector biology and control. Geneva 2123364

[CR54] Williams AB (2013). Residue analysis of organochlorine pesticides residue in water and sediments from Agboyi Creek, Lagos. Afr J Sci Technol.

[CR55] Yanez L, Ortiz-Perez D, Batres LE, Borja-Aburto VH, Diaz-Barriga F (2002). Levels of dichlorodiphenyltrichloroethane and deltamethrin in humans and environmental samples in Malarious areas of Mexico. Environ Res Sect A.

[CR56] Yang R, Ji G, Zhoe Q, Yaun C, Shi J (2005). Occurrence and distribution of organochlorine pesticides (HCH and DDT) in sediments collected from East China Sea. Environ Int.

[CR57] Zheng X, Liu X, Liu W, Jiang G, Yang R (2009). Concentrations and source identification of organochlorine pesticides (COCs) in soils from Wolong Natural Reserve. Chin Sci Bull.

[CR58] Zoumis T, Schmidt A, Grigorova L, Calmano W (2001). Contaminants in sediments: remobilisation and demobilization. Sci Total Environ.

